# Endometrial caspase 1 and interleukin-18 expression during the estrous cycle and peri-implantation period of porcine pregnancy and response to early exogenous estrogen administration

**DOI:** 10.1186/1477-7827-8-33

**Published:** 2010-04-09

**Authors:** Morgan D Ashworth, Jason W Ross, Daniel R Stein, Frank J White, Udaya W DeSilva, Rodney D Geisert

**Affiliations:** 1 Department of Animal Science, Oklahoma State University, Stillwater, OK 74078, USA; 2 Department of Animal Science, Cameron University, Lawton, OK 73505, USA; 3 Divison of Animal Sciences, University of Missouri, Columbia, MO 65211, USA; 4 Iowa State University, College of Agriculture and Life Sciences, Department of Animal Science, Ames, IA 50011, USA; 5 Yale University, Yale School of Medicine, Department of OBGYN, Reproductive Immunology Unit, New Haven, CT 06510, USA

## Abstract

**Background:**

The role for endometrial secretion of cytokines during the establishment of pregnancy in a number of mammals is well established. The current study determined endometrial expression of caspase 1 (CASP1) and interleukin-18 (IL18) during the estrous cycle and early pregnancy, and following early estrogen administration, which induces conceptus loss during early development in pigs.

**Methods:**

Gilts were hysterectomized on either D 0, 5, 10, 12, 15 and 18 of the estrous cycle, or D 10, 12, 15 or 18 of pregnancy. The abundance of endometrial CASP1 mRNA was unaffected by day of the estrous cycle, however there was a 6 and 10-fold increase in expression on D 15 and 18 of pregnancy. Endometrial expression of IL18 mRNA increased 5-fold between D 10 to 18 in cyclic and pregnant gilts. Total recoverable IL18 in uterine flushings was greater in pregnant compared to cyclic gilts on D 15 and 18.

In the second experiment, mated gilts were treated with either corn oil (CO) or estrogen (E) on D 9 and 10 and hysterectomized on either D 10, 12, 13, 15 or 17 of pregnancy. The current study localizes the presence of CASP1 to the epithelial layer of the endometrium for the first time. Further, a day × treatment interaction was detected for endometrial CASP1 mRNA and protein abundance as E stimulated an earlier increase on D 13 compared to CO gilts. Although IL18 mRNA expression remained unaltered from the E treatment, protein abundance was significantly attenuated on D 15 and 18 in response to E treatment.

**Conclusions:**

Endometrial expression of CASP1 and IL18 is associated with establishment of pregnancy in pigs. Alteration of CASP1 and IL18 following premature exposure of the uterus to estrogen during early pregnancy may contribute to conceptus loss between Days 15 to 18 of pregnancy.

## Background

The noninvasive attachment of the porcine conceptuses forms an epitheliochorial type of placentation through trophoblast adhesion to the extracellular glycocalyx present on the apical surface microvilli of the uterine luminal epithelium [1,2]. Following removal of anti-adhesive factors expressed on the uterine surface epithelium [3], trophoblast attachment to the uterine luminal epithelium involves a coordinated program of events in the progesterone stimulated uterine environment that are mediated by actions of conceptus estrogen secretion in the pig [4]. Trophoblast attachment to the uterine surface in the pig immediately follows the rapid elongation of the trophoblast and the acute increase of conceptus estrogen synthesis on Day 12 of pregnancy [5]. Release of estrogen by the elongating porcine conceptuses will induce endometrial receptivity for placental attachment to the uterine surface [6-8].

Transient release of estrogen during the period of rapid trophoblast elongation is concomitant with conceptus release of the proinflammatory cytokine, interleukin-1β (IL1B), which has been proposed to serve as the initial stimulus for conceptus trophoblast elongation and attachment to the uterine surface in the pig [9]. Furthermore, Ross *et al*. [9] demonstrated that the peri-implantation porcine conceptuses secrete the greatest amount of IL1B into the uterine lumen during trophoblast elongation and subsequent attachment of the filamentous conceptuses to the uterine epithelium between Days 12 to 15 which decreases to its nadir levels by Day 18 of gestation.

The decline in conceptus IL1B secretion suggests that another closely related cytokine may function at the conceptus and maternal uterine surface interface to continue regulation of the immunological interactions necessary for establishment of pregnancy in the pig. Interleukin 18 (IL18), formerly known as interferon-γ inducing factor [10], is a member of the IL-1 family of pro-inflammatory cytokines believed to play a significant role in implantation. Implantation in the mouse requires the presence of IL18 at the maternal fetal interface as indicated by abortion-prone mice which have suppressed IL18 secretion [11]. In contrast, mice that do not experience abortion produce elevated concentrations of IL18 at the maternal fetal interface suggesting a Th_1_/Th_2 _divergence. The Th_1_/Th_2 _paradigm involves the activation of naïve Th_0 _cells allowing the capability to diverge toward Th_1 _or Th_2_characteristics. The Th_1 _secretes cytokine patterns towards INFG, IL2 and IL18, where as Th_2 _secretes cytokines such as IL4, IL10, and IL6 that are usually associated as pro-pregnancy. The precise signal which determines the percent of Th_1 _versus Th_2 _is currently unkown.

Both IL1B and IL18 are synthesized as biologically inactive precursor peptides which must be proteolyically cleaved to be secreted and exhibit biological activity [12]. Caspase 1 (CASP1) cleaves and activates the pro-IL1B and pro-IL18 [12,13]. Conceptus secretion of interferon γ (IFNG) increases immediately following trophoblast elongation in the pig [14], suggesting that the conceptuses may induce endometrial expression of *IL18 *to assist in development and placental attachment during early pregnancy.

Exposure of gilts to estrogen before Day 12 of pregnancy causes conceptus degeneration following Day 15 of gestation [15]. Premature exposure of the uterine environment to estrogen prior to conceptus secretion in the pig alters the timing of endometrial gene expression during the period of placental attachment [16]. Our laboratory has previously reported that early estrogen exposure alters endometrial prostaglandin-endoperoxide synthase expression [17] and the insulin-like growth factor system [18]. The possible role of estrogen in regulating endometrial expression of *IL18 *and *CASP1 *has not been investigated.

The current study was undertaken to evaluate endometrial expression of *IL18 *and *CASP1 *during the estrous cycle and the period of early conceptus development and implantation in pigs. Furthermore, a second study investigated endometrial expression of *IL18 *and *CASP1 *following early exposure of the uterus to estrogen in pregnant gilts which causes adverse affects on conceptus development and survival in pigs.

## Methods

### Animals

Research was conducted in accordance with the Guiding Principles for Care and Use of Animals promoted and approved by the Oklahoma State Institutional Care and Use Committee. Crossbred cycling gilts of similar age (8-10 mo) and weight (100-130 Kg) were checked twice daily for estrus (Day 0) with intact males. Gilts assigned to be bred were naturally mated with fertile crossbred boars at first detection of estrus, and subsequently at 12 and 24 h post-estrus detection.

### Experiment I: Endometrial caspase-1 and IL18 expression in cyclic and pregnant gilts

Gilts were hysterectomized through midventral laparotomy on either Days 0, 5, 10 12, 15 or 18 of the estrous cycle (n = 24) or days 10, 12, 15 and 18 of pregnancy (n = 16) as previously described [19]. Following induction of anaesthesia with 1.8 ml i.m. administration of a cocktail consisting of 2.5 ml cocktail (Xylazine: 100 mg/ml: Miles Inc., Shawnee Mission, KS) and 2.5 ml Vetamine (Ketamine HCl: 100 mg/ml Molli Krodt Veterinary, Mundelein, IL) in 500 mg of Telazol (Tiletamine HCl and Zolazepum HCl: Fort Dodge, Syracuse, NE), anesthesia was maintained with a closed circuit system of halothane (5% flurothane) and oxygen (1.0 liters/min). The uterus was exposed via midventral laparotomy and the uterus and ovaries excised. Uterine horns were injected with 20 mL phosphate buffered saline (PBS, pH 7.4) via the isthmus and flushings were recovered in a petri dish. Conceptuses were removed from flushings, conceptus morphology was assessed and recorded and snap frozen in liquid N_2_, and stored at -80°C until utilized for extraction of total RNA. Uterine flushings were centrifuged (1000 × g, 10 min, 4°C), supernatant collected and uterine flushings free from debris were stored at -20°C. Endometrial tissue was removed from the antimesometrial side of the uterine horn, immediately snap frozen in liquid nitrogen and stored at -80°C until utilized for extraction of total RNA.

### Fixation of uterine tissue

Endometrial tissue sections (~1.0 cm) were excised from the bottom 40 cm of the uterine horn which was ligated prior to uterine flushing with PBS. Tissue sections were placed in freshly prepared 4% paraformaldehyde in PBS (pH 7.2) and gently agitated at r.t. for 24 h. Solution was replaced with 70% EtOH (v/v in H_2_0), gently agitated for an additional 24 h. Fixed endometrial tissue was dehydrated in a series of graded ethanol changes followed by xylene, and then embedded in Paraplast-Plus (Oxford Labware, St. Louis, MO).

### Experiment II: Endometrial caspase-1 and IL18 gene and protein expression following early exposure of pregnant gilts to estrogen

Bred gilts were assigned randomly to one of the following treatment groups: Control (n = 20), i.m. injection of corn oil (CO) (2.5 ml) on days 9 and 10 of gestation or estrogen (E) (n = 20), 5 mg i.m. injection of estradiol cypionate (A.J. Legere, Scottsdale, AZ) on days 9 and 10 of gestation. Gilts in the CO and E treatment groups were hysterectomized on either day 10, 12, 13, 15 and 17 of gestation as previously described in experiment 1. Uterine horns were flushed with 20 ml PBS, conceptuses were collected, flushings centrifuged to remove cellular debris, and the supernatant stored at -20°C. Conceptuses were visually evaluated to determine if they were normal (spherical to filamentous morphology) on days 10 and 12 of pregnancy and were intact filamentous conceptuses collected on days 13, 15 and 17 of pregnancy. Endometrial tissue was harvested from the uterine horn and either fixed for *in situ *hybridization or snap frozen in liquid nitrogen and stored at-80°C until utilized for extraction of total RNA.

### RNA extraction

Total RNA was extracted from endometrial and conceptus tissues using RNAwiz™ reagent (Ambion, Inc. Austin, TX). Approximately 0.5 g of endometrial tissue was homogenized in 5.0 mL of RNAwiz using a Virtishear homogenizer (Virtis Company Inc., Gardiner, NY). RNA was resuspended in nuclease free water and stored at -80°C. Total RNA was isolated from conceptus pools collected from the uterine horns on Days 10, 12, 15 and 18 of pregnancy using RNAwiz™ reagent (Ambion, Inc. Austin, TX) as previously described by [20]. Total RNA was quantified with a Nanodrop^® ^spectrophotometer at an absorbance of 260 nm and purity was verified using the 260/280 ratio.

### Quantitative 1-step RT-PCR

Quantitative analysis of endometrial *CASP1 *and *IL18 *mRNA were analyzed using quantitative real time reverse polymerase chain reaction (RT-PCR) as previously described by our laboratory [17,21]. The PCR amplification was performed in a reaction volume of 15 μl using an ABI PRISM 7500 Sequence Detection System (PE Applied Biosystems, Foster City, CA). The transcripts were evaluated using dual labeled probes with 6-Fam (5' reporter dye), and TAMRA (3' quenching dye). Primer and probe sequences for the amplification of *CASP1 *and *IL18 *(Table 1) were generated from porcine sequences obtained using the NCBI genebank database. Total RNA (100 ng) was assayed in duplicate using thermocycling conditions for one-step cDNA synthesis of 30 min at 48°C and 95°C for 10 min, followed by 45 repetitive cycles of 95°C for 15 sec and 60°C for 1 min. Ribosomal 18S RNA was assayed in each sample to normalize RNA loading.

**Table 1:** PCR primer and probe sequences used for quantitative RT-PCR of endometrial *CASP1 *and *IL18 *mRNA expression

Gene	Forward Primer/Reverse Primer/Probe	GeneBank Accession
**Caspase-1**		
	*Forward *5'-CAA CAC TTC ACC CAC CAG TTC TC-3'	NM214162
	*Reverse *5'-TCC ATA AAT GTG GCC GAG GTC TAC-3'	
	*Probe *5'-TTC TGG CAA GAT GGG TCC TGG CTT CAC CAA-3'	
**IL18**		
	*Forward *5'-ATG GCT GCT GAA CCA GAA GA-3'	NM213997
	*Reverse *5'-TGG TCG TTC AGA TTT CGT ATG ATT-3'	
	*Probe *5'-CCT GGA ATC GGA TTA CTT TGG CAA GC-3'	

Using the comparative C_T _method [17], relative quantification and fold gene expression difference between treatment and day were determined for the conceptus and endometrial samples. Differences in mRNA expression of *CASP1 *and *IL18 *were determined by subtracting target C_T _of each sample from its respective ribosomal 18S C_T _value, which provides the sample ΔC_T _value. Calculation of the ΔΔC_T _involves using the highest sample ΔC_T _value as an arbitrary constant to subtract from all other ΔCT sample values. The mean value of Day 10 pregnant and Day 5 cyclic (highest ΔC_T_; lowest gene expression for *CASP1 *and *IL18 *genes respectively) were used as baselines for *CASP1 *and *IL18 *to set the baseline for comparing differences in ΔC_T _values across all days when comparing the cyclic animals to the pregnant animals in Experiment I.

The mean value of Day 12 estrogen treated and Day 12 control for *CASP1 *and *IL18 *genes respectively, were used to set the baseline for comparing differences in ΔC_T _values for the endometrium in Experiment II. Fold differences in gene expression of the target gene are equivalent to 2^-ΔΔCT^.

### Enzyme-linked CASP1 and IL18 competitive binding assay

Total luminal content of CASP1 in the uterine flushings was quantified using a commercial ELISA kit (BenderMed Burlingame, CA) in accordance with manufacturer's specifications. Samples were analyzed in duplicate with a single assay. The intra-assay coefficient of variation for the assay was 5.6%. Total luminal content of IL18 in the uterine flushings was quantified using a commercial ELISA kit (BenderMed Burlingame, CA) in accordance with manufacturer's specifications. Samples were analyzed in duplicate with a single assay. The intra-assay coefficient of variation for the assay was 5.1%.

### *In situ *hybridization

*CASP1 *mRNAs were localized in paraffin-embedded porcine uterine tissue by *in situ *hybridization using methods previouslydescribed [22]. Briefly, deparaffinized, rehydrated, and deproteinateduterine cross-sections (5 μm) were hybridized with radiolabeledantisense or sense porcine caspase-1 cRNA probes synthesizedby *in vitro *transcription with [α-^35^S] uridine 5-triphosphate(PerkinElmer Life Sciences). After hybridization, washes, andRNase A digestion, autoradiography was performed using NTB-2liquid photographic emulsion (Eastman Kodak, Rockchester, NY). Slides were exposed at 4°C for 6 days, developed in Kodak D-19developer, counterstained with Harris' modified hematoxylin(Fisher Scientific, Fairlawn, NJ), dehydrated, and protectedwith coverslips.

### Statistical analysis

Data were analyzed by least squares ANOVA using the Proc Mixed model of the Statistical Analysis System (SAS Institute Inc., Cary, NC). The statistical model used to evaluate endometrial *CASP1 *and *IL18 *mRNA expression and uterine flushing content of IL18 and CASP1in Experiment I included the effects of day (0, 5, 10, 12, 15, and 18), reproductive status (cyclic, pregnant), and the day × status interaction. If the status, day, or day × status interactions were significant, (*P *< 0.05), means were separated by using the PDIFF option of SAS. Additionally, due to unequal variances of CASP1 and IL18 protein in the uterine flushings of the cyclic and pregnant gilts, the data were log transformed.

The statistical model used to evaluate endometrial *CASP1 *and *IL18 *mRNA expression and uterine flushing content in Experiment II included the effects of day (10, 12, 13, 15, and 17), treatment (CO, E), and the day × treatment interaction. If the day, treatment or day by treatment interaction was significant, (*P *< 0.05), treatment means were then separated by using the PDIFF option of SAS. Additionally, due to unequal variances of CASP1 and IL18 protein in the uterine flushings of the pregnant gilts, the data was log transformed for the statistical analysis.

## Results

### Experiment I: Cyclic and pregnant gilts

#### Conceptus IL18 and Caspase-1 mRNA expression

Expression of *IL18 *mRNA (data not shown) was not detected by RT-PCR in any of the developing porcine conceptuses collected on days 10 to 18 of pregnancy. Despite the lack of IL-18 mRNA expression, *CASP1 *mRNA was present in the developing conceptuses from days 10 to 18 of pregnancy. Conceptus expression of *CASP1 *mRNA (data not shown) was similar on Days 10, 12, increasing 2-fold (P < 0.05) on Days 15 and 18 of pregnancy.

#### Endometrial IL18 mRNA and protein expression

**Figure 1:**
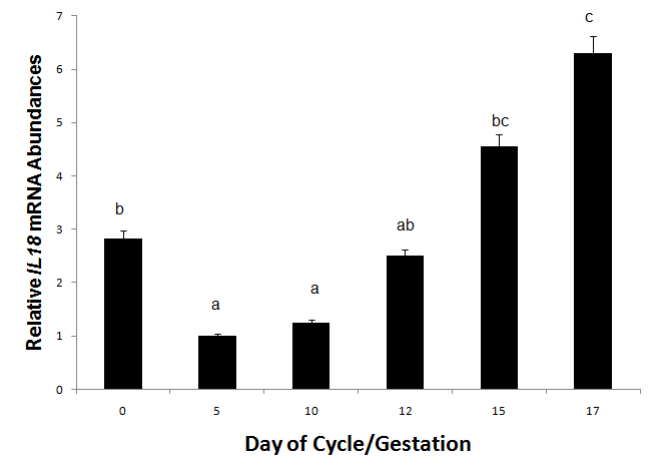
**Relative mRNA abundance in endometrial *IL18 *mRNA expression from gilts (n = 4/day/status) on days 0, 5, 10, 12, 15, and 18 of the estrous cycle (open bars) and days 10, 12, 15 and 18 of pregnancy (black bars)**. Abundance of mRNA was calculated from the real-time PCR analysis as described in *Methods and Materials*. A significant day effect on relative mRNA units (mean ± SEM) was identified for endometrial *IL18 *(*P *< 0.05). Days without a common superscript represent a statistical difference.

Endometrial *IL18 *mRNA expression was not affected by status, but a significant day effect (*P *< 0.05) was detected. Endometrial *IL18 *mRNA increased on Days 15 and 18 of the estrous cycle and pregnancy (Figure 1).

**Figure 2:**
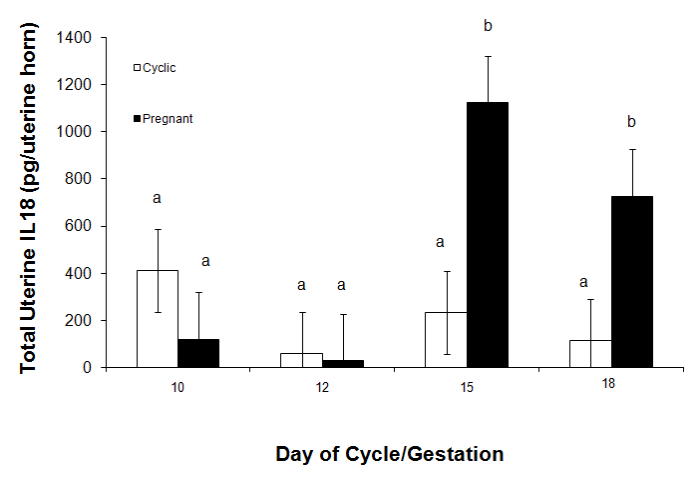
**Relative content of total IL18 (pg) in uterine flushing collected from gilts (n = 4/day/status) on days 0, 5, 10, 12, 15 and 18 of the estrous cycle (open bars) and days 10, 12, 15 and 18 of pregnancy (black bars)**. A day × status interaction (*P *< 0.05) was detected for total IL18. Bars without a common superscript represent a statistical difference.

A day × status interaction (*P *< 0.05) was detected for recoverable IL18 in uterine flushings of cyclic and pregnant gilts. The uterine luminal content of IL18 was similar across all days of the estrous cycle. However, although the uterine luminal content of IL18 on Days 10 and 12 of pregnancy were comparable to cyclic gilts, there was an approximate 5-fold increase in IL18 on Days 15 and 18 pregnancy compared to uterine flushings collected on Days 15 and 18 of the estrous cycle (Figure 2).

#### Endometrial Caspase-1 mRNA and protein expression

**Figure 3:**
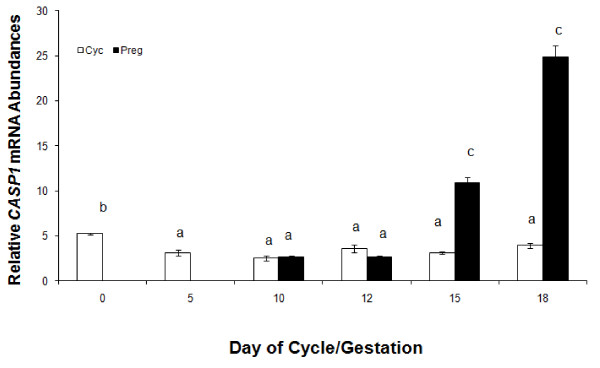
**Relative mRNA adundance in endometrial caspase-1 mRNA expression from gilts (n = 4/day/status) on days 0, 5, 10, 12, 15, and 18 of the estrous cycle (open bars) and days 10, 12, 15 and 18 of pregnancy (black bars)**. Abundance of mRNA was calculated from the real-time PCR analysis as described in *Methods and Materials*. A significant day × status interaction on relative mRNA units (mean ± SEM) was identified for endometrial caspase-1 (*P *< 0.05). Bars without a common superscript represent a statistical difference.

**Figure 4:**
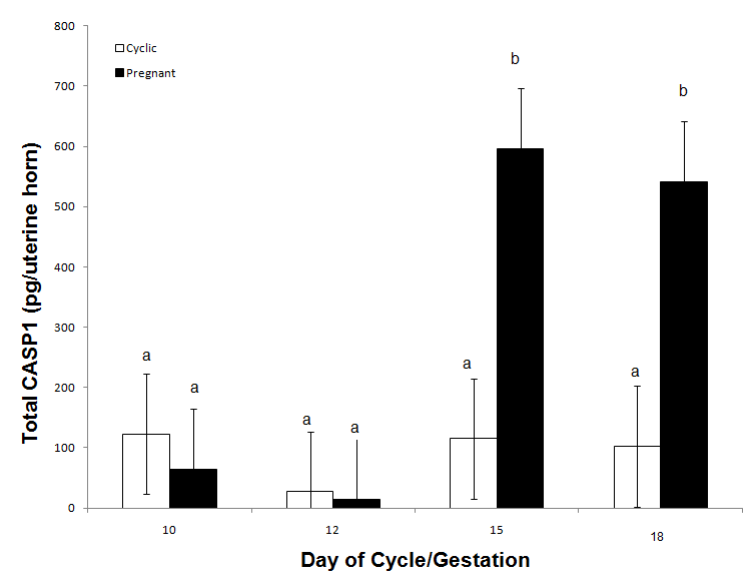
***In situ *hybridization analysis of *caspase-1 *mRNA expression in porcine endometrium**. Protected transcripts in endometrium from day 15 of pregnancy was visualized by liquid emulsion autoradiography and imaged under bright-field and dark-field illumination. Endometrial sections from E treated section on Day 12 of pregnancy was hybridized with radiolabeled sense cRNA probe to serve as a negative control. *Caspase-1 *mRNA expression is abundant in the luminal (L) and glandular (G) epithelium but low to absent in the stroma (S). 4× Objective and 10× eyepiece.

Quantitative RT-PCR analysis of endometrial *CASP1 *mRNA in cyclic and pregnant gilts detected a day × status interaction (*P *< 0.05). Endometrial expression of *CASP1 *mRNA was approximately 3 and 5-fold greater in pregnant compared to cycling gilts on Days 15 and 18, respectively (Figure 3). *In situ *hybridization analysis to localize cellular mRNA expression demonstrated that *CASP1 *mRNA expression was localized to the uterine luminal and glandular epithelia on Day 15 of pregnancy (Figure 4).

**Figure 5:**
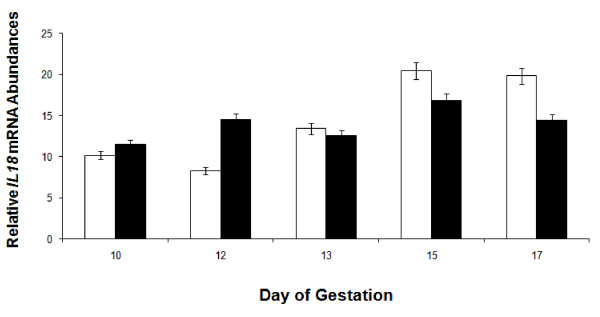
**Relative content of total caspase-1 protein (pg) in uterine flushings from gilts (n = 4/day/status) on days 10, 12, 15 and 18 of the estrous cycle (open bars) and pregnancy (black bars)**. Content of caspase-1 in the uterine luminal flushings of cyclic and pregnant gilts exhibited a day by status interaction (*P *< 0.05). Bars without a common superscript represent a statistical difference.

A day × status interaction (*P *< 0.05) was detected for recoverable CASP1 protein in uterine luminal flushings of cyclic and pregnant gilts (Figure 5). Uterine luminal content of CASP1 was low in all days of estrous cycle and days 10 and 12 of pregnancy (27 to 123 pg); however, during pregnancy there was a 5-fold increase in the luminal content of CASP1on Days 15 and 17 (595 and 541 pg, respectively).

### Experiment II: Early exposure of pregnant gilts to estrogen

#### Conceptus Development

Conceptuses with normal morphology were recovered in uterine flushings collected from CO gilts on all days of pregnancy evaluated visually (Days 10, 12, 13, 15, and 17). Intact and normal spherical, ovoid, and filamentous conceptuses were collected from E-treated gilts on Days 10, 12 and 13 of gestation; however, the filamentous conceptuses were in various stages of fragmentation when collected on Days 15 and 17 of gestation.

#### Endometrial IL18 mRNA and protein expression

**Figure 6:**
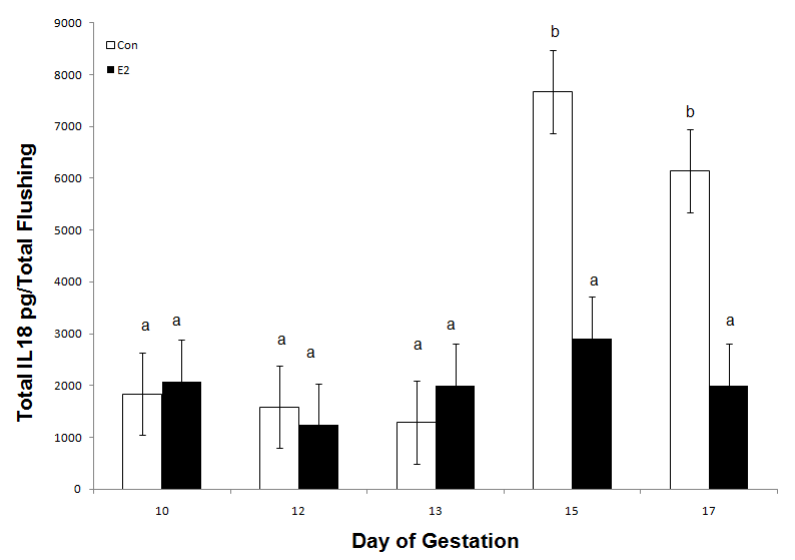
**Relative mRNA abundance in endometrial *IL18 *mRNA expression of control (open bars) and estrogen (black bars) treated gilts (n = 4/day/treatment) on days 10, 12, 13, 15 and 17 of pregnancy**. Abundance of mRNA was calculated from the real-time PCR analysis as described in *Methods and Materials*. No significant main effects or interaction were observed on relative mRNA units (mean ± SEM) for endometrial *IL18 *(P > 0.05).

**Figure 7:**
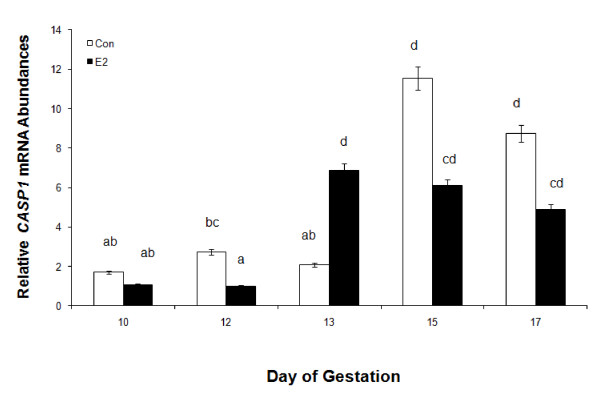
**Relative content of total IL18 (pg) in uterine flushing collected from control (open bars) and estrogen (black bars) treated gilts (n = 4/day/treatment) on days 10, 12, 13, 15 and 17 of pregnancy**. A day × treatment interaction (*P *< 0.05) was detected. Bars without a common superscript represent a statistical difference.

There was no effect of E-treatment (*P *> 0.05) on endometrial *IL18 *mRNA abundance (Figure 6). However, a day × treatment interaction (*P *< 0.05) was detected for the uterine luminal content of IL18 collected in uterine flushings of CO- and E-treated pregnant gilts (Figure 7). Recoverable IL18 in uterine flushings of CO gilts significantly increased on Days 15 and 17 of pregnancy. However in contrast to CO-treated gilts, the uterine luminal content of IL18 did not increase on Days 15 and 17 in E-treated gilts.

#### Endometrial CASP1 mRNA and protein expression

**Figure 8:**
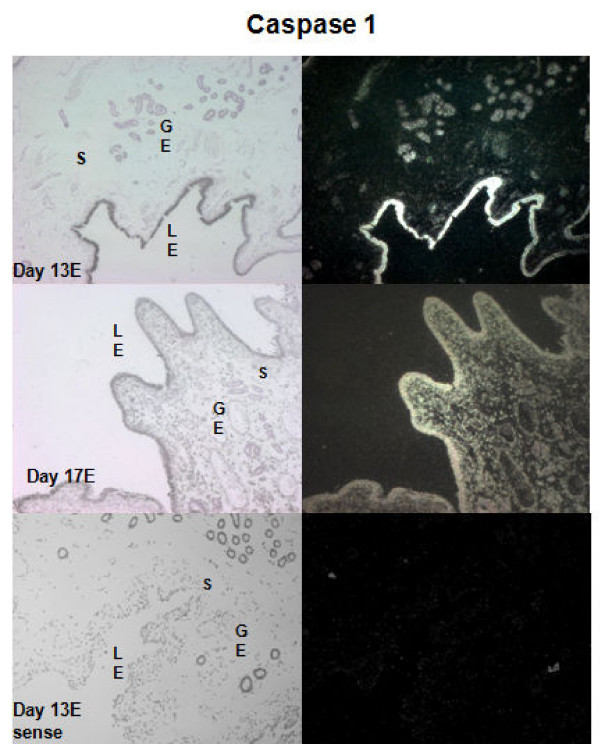
**Relative mRNA abundance in endometrial caspase-1 mRNA expression of control (open bars) and estrogen treated (black bars) gilts (n = 4/day/treatment) on days 10, 12, 13, 15 and 17 of pregnancy**. Abundance of mRNA was calculated from the real-time PCR analysis as described in *Methods and Materials*. A significant day by treatment interaction was observed on relative mRNA units (mean ± SEM) was identified for endometrial *caspase-1 *(P < 0.05). Bars without a common superscript represent a statistical difference.

A day × treatment interaction (*P *< 0.05) was detected for endometrial *CASP1*mRNA abundance in E compared to CO gilts. Endometrial *CASP1 *mRNA abundance was lowest in E and CO gilts on Days 10 and 12 of gestation (Figure 8). However, E stimulated a 3-fold earlier increase in *CASP1 *expression on Day 13 compared to CO gilts. The increase in *CASP1 *abundance levels remained elevated in both E-treated gilts and CO-treated gilts on Days 15 and 17 of gestation.

**Figure 9:**
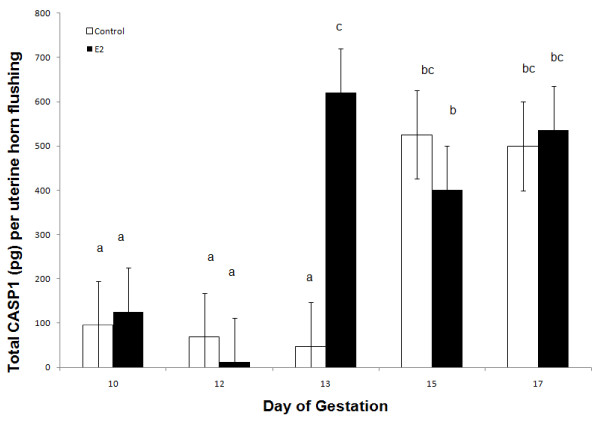
**Relative content of total caspase-1 protein (pg) in uterine flushing collected from control (open bars) and estrogen treated (black bars) gilts (n = 4/day/treatment) on days 10, 12, 13, 15 and 17 of pregnancy**. A day × treatment interaction (*P *< 0.05) was detected. Bars without a common superscript represent a statistical difference.

A day × treatment interaction (*P *< 0.05) was detected for CASP1 protein in uterine flushings of CO- and E-treated gilts (Figure 9). The uterine luminal content of CASP1 was increased in both CO- and E-treated gilts on Days 15 and 17 of pregnancy. However, E-treated gilts exhibited a premature increase in uterine luminal CASP1 content on Day 13 (650 pg) compared to CO-treated gilts (50 pg).

## Discussion

The peri-implantation period (Days 10 to 17) accounts for the greatest embryonic loss in pigs [23]. Between Days 11 to 12 of gestation the porcine conceptuses will begin to synthesize and secrete estrogen into the uterine lumen signaling maternal recognition and establishment of pregnancy [24]. On Day 13 of gestation conceptus attachment to the uterine surface epithelium is initiated by trophectoderm of post-elongated porcine conceptuses. The glycocalyx on the microvillus luminal surface epithelium of the uterus serves as an intermediate attachment substrate for trophectoderm to interdigitate with the uterine luminal epithelium [6]. Conceptus attachment to the uterine surface and secretion of estrogen is a critical period of temporal endometrial changes in gene expression that allows the establishment of pregnancy [16,25].

Ross *et al*. [9] demonstrated that peri-implantation porcine conceptuses begin to secrete large amounts of IL1B into the uterine lumen during the transition from spherical to filamentous morphology on Day 12 of gestation with peak values during trophoblast elongation and values declining to nadir levels by Day 18 of gestation. Both pro-IL1B and pro-IL18 are substrates for CASP1 cleavage which allows activation and cellular release of these cytokines [12]. The current study provides evidence that porcine conceptuses express *CASP1 *mRNA throughout the early stages of development which would provide for the release of conceptus IL1B into the uterine lumen. The lack of any large change in conceptus *CASP1 *mRNA production from Day 12 to 18 of pregnancy indicate that changes in conceptus *IL1B *mRNA expression are responsible for the increase and decline of IL1B in the uterine lumen [9]. However, in contrast to IL1B, IL18 can be stored in the cell and its biological activity is thus mainly due to pro-IL18 processing by CASP1 [12]. In vitro studies using insect cells, which do not produce endogenous CASP1, demonstrated that porcine IL18 lacks a functional signal peptide and requires CASP1 to release and secrete significant amounts of functional mature IL18 [26].

The pregnancy specific increase in endometrial CASP1mRNA expression and protein production is consistent with a role of CASP1 that increased release of IL18 into the uterine lumen on Day 15 and 18 of pregnancy. The early stages of porcine conceptus development did not express IL18 mRNA indicating that the endometrium is the source of IL18 in the uterine lumen. Although results of the present study cannot establish the conceptus product that stimulates IL18 secretion into the uterine lumen, IL18 release is temporally associated with the increase in CASP1 mRNA expression and protein production during Day 15 and 18 of pregnancy. Our results suggest that an increased release of IL18 from the endometrium induced by the porcine conceptuses, shifts the interaction of the type I cytokines from the conceptus IL1B [9,17] to an endometrial IL18 after Day 12 of pregnancy which may be necessary for conceptus attachment to the uterine surface and maintenance of pregnancy in the pig. IL18, which has structural similarities to IL1B, is involved with modulation of the immune system through induction of INFG [27]. Although similar in structure and action to IL1B, IL18 binds a unique IL18 receptor and functions independently of the nuclear factor kappa B pathway [28]. IL18 expressed by the human endometrial epithelia has been suggested to regulate maternal-embryo interplay during establishment of pregnancy [29].

Endometrial production of IL18, also known as interferon inducing factor due to its ability to induce interferon gamma, may play are role in regulating the uterine immune system through a possible influence on the increased secretion of IFNG by pig conceptuses between Days 15 and 18 of gestation [14,30]. Although speculative, the increased endometrial expression of CASP1 and release of IL18 into the uterine lumen may induce expression and secretion of IFNG by conceptuses to modulate the maternal immune system at the interface between trophectoderm and uterine luminal epithelium. Further studies are necessary to demonstrate that IL18 directly stimulates porcine conceptus INFG production.

Our laboratory has detected expression of the immune modulator, chemokine ligand 9 (CXCL9) in porcine endometrium and determined that *CXCL9 *mRNA expression increases during the window of implantation in pigs (Ashworth, unpublished data). IL18 has been reported to increase CXCL9 expression by 10-fold in human macrophages and there is a synergistic 50-fold increase in *CXCL9 *in response to the combination of IL18 and IL-12 [31]. Therefore, it is plausible that IL18 may serve to stimulate induction of *CXCL9 *in the endometrium during early pregnancy in pigs.

It has been well established that exposure of pregnant gilts to exogenous estrogen 48 h prior to when it is normally secreted by conceptuses on Days 11 and12 results in death and fragmentation of conceptuses between Days 15 to 18 of gestation [15,23]. Conceptus degeneration is correlated with the spatiotemporal loss of the glycocalyx of microvilli on the endometrial luminal epithelium [32]. Premature exposure of pregnant gilts to estrogen advances the profile of endometrial gene expression compared to normal pregnancy [33]. The early expression of endometrial CASP1 following premature estrogen exposure is consistent with expression of other gene markers following Day 12 of pregnancy. Endometrial expression of insulin-like growth factors and prostaglandin-endoperoxide synthase 2 during the peri-implantation period in pigs are advanced by 48 h due to premature estrogen exposure [17,18].

In the current study, early estrogen administration disrupts proper conceptus development in pregnant pigs which exhibited an alteration of endometrial IL18 secretion during the period of conceptus attachment to the uterine surface. Interestingly, although both CASP1 mRNA expression and protein production was advanced, the release of IL18 protein secretion into uterine lumen was significantly compromised during the peri-implantation period. Thus, despite a premature increase in endometrial *CASP1 *mRNA and protein expression, the release of IL18 into the uterine lumen became uncoupled in estrogen treated gilts. The mechanism by which IL18 secretion into the uterine lumen was inhibited in the presence of increased CASP1 is not known. The expression of *IL18 *mRNA in the endometrium was not altered by the early estrogen treatment suggesting either an inhibition of CASP1 cleavage of pro-IL18 or binding of IL18 to its soluble IL-18-binding protein prevent secretion and/or detection of IL18 in the uterine flushing [12]. IL-18-binding protein only binds to the biologically active IL-18 and blocks its interaction with cell surface receptors [12]. Thus, although there is an increase of CASP1 protein in the estrogen treated gilts, binding to its binding protein may inhibit its biological activity and our ability to measure IL-18 in the uterine flushing. Further studies are needed to investigate the changes in endometrial IL-18BP in the pig.

It is possible that the decrease in biologically active IL18 in uterine lumen of gilts treated with estrogen may contribute to a cascade of endometrial changes that result in the failure of conceptus development and implantation. Recurrent spontaneous abortion in mice is associated with decreased levels of IL18 at the embryo-maternal interface [11]. Early studies emphasized a strong role of IL18 as an interferon-γ inducing factor [10]. The failure of endometrial IL18 secretion in estrogen-treated gilts would be consistent with the lack of endometrial stromal expression of the Signal Transducers and Activators of Transcription 1 (*STAT1*) in estrogen treated pregnant gilts [30]. Conceptus IFNG, which stimulates endometrial *STAT1 *expression, was comprised in gilts treated with estrogen early in pregnancy. The combined results of our study and Joyce et al. [30] indicate that disruption of conceptus attachment following endocrine disruption with estrogen interferes with release of endometrial IL18 and induction of IFNG in developing conceptuses.

The current study establishes the normal profile for transcriptional and translational expression of *IL18 *and *CASP1 *in the endometrium during the porcine estrous cycle and early gestation. Results indicate that endometrial *CASP1 *mRNA and protein expression and secretion of IL18 from the endometrium are regulated by the presence of viable conceptuses in the uterine lumen. Furthermore, an uncoupling of endometrial CASP1 production and secretion of IL18 into the uterine lumen is temporally associated with conceptus degeneration following endocrine disruption of pregnant gilts with premature exposure to estrogen during early pregnancy.

## Competing interests

The authors declare that they have no competing interests.

## Authors' contributions

MDA carried out the molecular studies, conceived the study and its design, and drafted the manuscript. JWR participated with surgeries, carried out the *in situ *hybridization, participated in ELISA studies, and helped to draft the manuscript. DRS participated in surgeries. FJW participated with drafting the manuscript and statistical analysis. UWD participated with drafting the manuscript. RDG participated in conception of the study, and participated in its design and coordination and helped to draft the manuscript. All authors have read and approved the final manuscript.
